# Biochemistry Changes That Occur after Death: Potential Markers for Determining Post-Mortem Interval

**DOI:** 10.1371/journal.pone.0082011

**Published:** 2013-11-21

**Authors:** Andrea E. Donaldson, Iain L. Lamont

**Affiliations:** Department of Biochemistry, University of Otago, Dunedin, New Zealand; Laurentian University, Canada

## Abstract

Death is likely to result in very extensive biochemical changes in all body tissues due to lack of circulating oxygen, altered enzymatic reactions, cellular degradation, and cessation of anabolic production of metabolites. These biochemical changes may provide chemical markers for helping to more accurately determine the time since death (post-mortem interval), which is challenging to establish with current observation-based methodologies. In this study blood pH and changes in concentration of six metabolites (lactic acid, hypoxanthine, uric acid, ammonia, NADH and formic acid) were examined post-mortem over a 96 hour period in blood taken from animal corpses (rat and pig) and blood from rats and humans stored *in vitro*. The pH and the concentration of all six metabolites changed post-mortem but the extent and rate of change varied. Blood pH in corpses fell from 7.4 to 5.1. Concentrations of hypoxanthine, ammonia, NADH and formic acid all increased with time and these metabolites may be potential markers for post-mortem interval. The concentration of lactate increased and then remained at an elevated level and changes in the concentration were different in the rat compared to the human and pig. This is the first systematic study of multiple metabolic changes post-mortem and demonstrates the nature and extent of the changes that occur, in addition to identifying potential markers for estimating post-mortem interval.

## Introduction

Metabolites are small-molecule intermediates and products of metabolism that have multiple functions in normal growth and development of cells, including generation of cellular energy, serving as substrates for synthesis of macromolecules and cellular signalling. Metabolites can be endogenous molecules produced by the host, or exogenous molecules derived from chemicals in foods or produced by microbes [1]. The relative amounts of metabolites in a tissue provide information on cellular function and can change in response to endogenous variables [1]. Metabolic profiles in body fluids reflect endogenous changes and so changes that occur following death should manifest in altered metabolite profiles. The agonal period, supravital reactions (reactions occurring from the moment of death until cellular functions cease), leakage from cell degradation, and degradation of proteins should all be reflected in metabolite profiles. This means that analysis of metabolites from body fluids can provide insights into the changing biochemical environment post-mortem. 

Metabolic changes that occur post-mortem have been identified and attributed to the agonal period of anoxia, the continuation of biochemical changes in the early post-mortem period, and the distribution of easily diffusible substances between erythrocytes and plasma as well as between interstitial fluid, tissue cells, and the blood [2]. Antemortem blood pH is regulated to stay within the narrow range of 7.35 to 7.45. If the blood pH is alkaline (greater than 7.45) or acidic (less than 7) this can lead to death [3] so blood pH is one of the most regulated systems in the body. Blood pH is regulated by acid-base buffers such as carbonic acid and biocarbonate ion, which exert their influence principally through the respiratory system and the kidneys in order to control the acid-base balance [3]. In death, the body buffering system is not maintained and blood pH changes can occur [4].

 An early study reported that after death cardiac blood taken directly from 11 individual human remains showed a pH change from 7.0 to 5.5 by 20 hours post-mortem [5]. In the same study, blood collected from rat corpses showed a pH decrease from 7.35 at antemortem to 5.5 by 96 hours post-mortem. 

A lower blood pH must reflect the accumulation of acidic metabolites. Lactic acid is produced by lactate dehydrogenase from pyruvate via anaerobic glycolysis in skeletal muscle, liver and red blood cells when insufficient oxygen is available for pyruvate to enter the citric acid cycle. This process occurs naturally in muscle tissue during exercise and in normal metabolism inside red blood cells. The normal serum lactate concentration is 0.5-2.2 mmol.L^-1^. Circulating lactate is normally oxidised to pyruvate through the actions of lactate dehydrogenase (LDH) after being taken up by monocarboxylate transport proteins (MCTs) that are differentially expressed in actively respiring cells and tissues [6]. In the only recent study of lactate concentration in blood post-mortem, lactate in human heart blood increased 20-fold by one hour after death and 50-70 fold by 24 hours [2]. 

Formate (methanoic acid) is the simplest carboxylic acid. Formate is the toxic metabolite of both methanol and formaldehyde and is normally present in mammals in low concentrations (0.12-0.28 mmol.L^-1^) [7] following catabolism of several amino acids including serine, glycine, histidine, and tryptophan [8] as well as catabolism of methanol from external sources such as diet [9]. In humans formate is rapidly oxidised to carbon dioxide by the liver [9], to prevent toxic effects in the brain and eyes due to an inhibition of the cytochrome oxidase a protein complex in the respiratory chain in mitochondria [10]. In the only study carried out to date, the concentrations of formate are elevated in the blood of putrefied (5-44 days) post-mortem human cases (5 mmol.L^-1^) compared to non-putrefied (1-14 days) post-mortem human cases (0.86 mmol.L^-1^) [11]. It was suggested that the reason for this increase was bacterial action on decomposition of lipids and proteins [11]. 

Nicotinamide adenine dinucleotide (NAD^+^) and its reduced form NADH are substrates that have central roles in cellular metabolism and energy production as hydride-accepting and hydride-donating coenzymes in the citric acid cycle. During hypoxic conditions NAD^+^ cannot be regenerated from NADH which accumulates due to anaerobic glycolysis and peroxisomal catabolism of fatty acids [4,12]. However, NADH has not been examined post-mortem. 

Nitrogenous compounds are also expected to change post-mortem as degrading tissues undergo nucleotide and protein catabolism. Ammonia is formed in nearly all tissues and organs by normal amino acid and nucleotide catabolism. Antemortem, ammonia is rapidly removed from circulation by the liver where it is converted to urea, or removed from skeletal muscle by the glucose-alanine cycle, leaving only traces (11.6-32.2 μmol.L^-1^) in the bloodstream [13]. During decomposition of a corpse, the ammonia produced from amino acids, nucleotide and tissue degradation may accumulate over time as it is not removed by the liver, although as ammonia is volatile, it may also be lost from the corpse into the environment. In the only study relating PMI and ammonia concentrations, the concentration of ammonia in plasma was reported to increase after death with a rapid rise after 8 hours [14]. However, the highest ammonia concentration obtained and the time frame of the experiment was not reported. 

Hypoxanthine is an intermediate in the purine catabolism pathway. Antemortem it is oxidised by xanthine oxidase to uric acid, the end product of purine metabolism in humans. The xanthine oxidase enzyme in mammals is present mainly in the liver and the small intestinal mucosa. Therefore because there is no xanthine oxidase in the blood, the end product of nucleotide catabolism in the bloodstream is hypoxanthine, which is taken up by the liver to be converted to uric acid and excreted. The normal concentration of hypoxanthine in blood is 0.5-11 μmol.L^-1^ [15,16]. During tissue hypoxia, hypoxanthine concentrations are raised in plasma, cerebrospinal fluid and urine [16-18] due to limited oxygen available as a co-factor for xanthine oxidase. There is evidence that the hypoxanthine concentration increases for the first 24 hours after death in both humans and animals [19,20] but detailed studies have not been carried out.

Uric acid is the end product of purine metabolism in humans [21]. It is formed by the liver and excreted by the kidneys (65-75%) and intestines (25-35%). Uric acid is a weak acid that circulates in plasma predominantly (98%) in the form of a sodium salt (urate) and is an excellent antioxidant, responsible for 2/3 of total plasma antioxidant capacity [21,22]. The normal concentration of uric acid in human plasma is 137-494 µmol.L^-1^. Uric acid concentration was described as being elevated in the blood of delayed traumatic deaths due to multiple organ insufficiency [23]. It has been proposed that the increase in uric acid concentration in delayed traumatic deaths is a pro-inflammatory marker to indicate necrosis and death –induced inflammation [23]. In other mammals such as rats, uric acid is degraded by uricase into allantoin and excreted so uric acid should remain stable post-mortem. Humans lack the uricase enzyme.

The aims of this study were to determine the changes in blood pH and concentrations of lactic acid, hypoxanthine, uric acid, ammonia, NADH and formate in blood post-mortem taken directly from the corpses of rats and pigs. Research was also conducted using blood from rats and humans that was stored in a tube over a period of 96 hours with pH and metabolites being measured at intervals, in order to identify blood metabolic changes that occurred as a result of tissue degradation.

## Results

### Changes in blood pH

**Figure 1:**
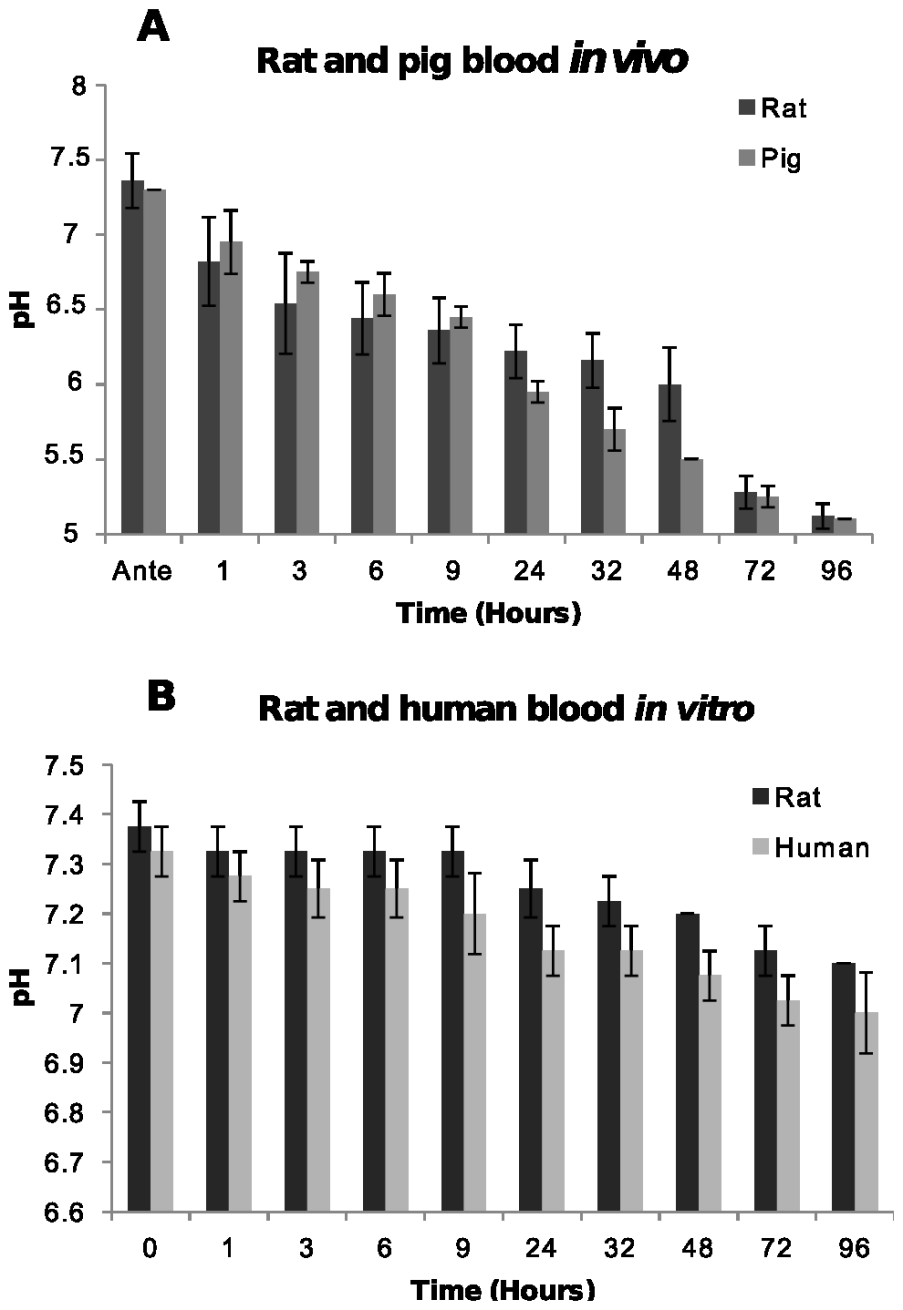
Blood pH. [A] In post-mortem blood from rat and pig corpses over 96 hours. The average pH of blood samples from five rat and two pig corpses at each time point are shown with standard deviations. [B] In human blood and rat blood stored in a tube. The average pH and standard deviations at each time point from two human and three rat blood samples are shown.

We first measured pH changes in post-mortem blood. Blood pH was measured for human and rat blood stored in EDTA tubes and for rat and pig blood stored in corpses (Figure 1). 

Rat and pig blood in corpses showed a decrease in blood pH from 7.45 antemortem to 6.1 after 24 hours and to pH 5.1 after 96 hours. The pH of human blood and post-mortem rat blood stored in a tube decreased very slightly compared to antemortem levels going from 7.4 to 7.1 over 96 hours (Figure 1 B). The rapid decrease in blood pH *in vivo* (Figure 1 A) is most likely related to the accumulation of metabolites and ions such as bicarbonate, carbon dioxide, hydrogen ions, di-hydrogen phosphate ions, and lactic acid building up in a corpse due to autolysis, which does not occur *in vitro*.

### Changes in lactate and formate

The carboxylic acids lactate and formate were examined to establish if the concentrations of these metabolites changed post-mortem, contributing to lower pH. 

**Figure 2:**
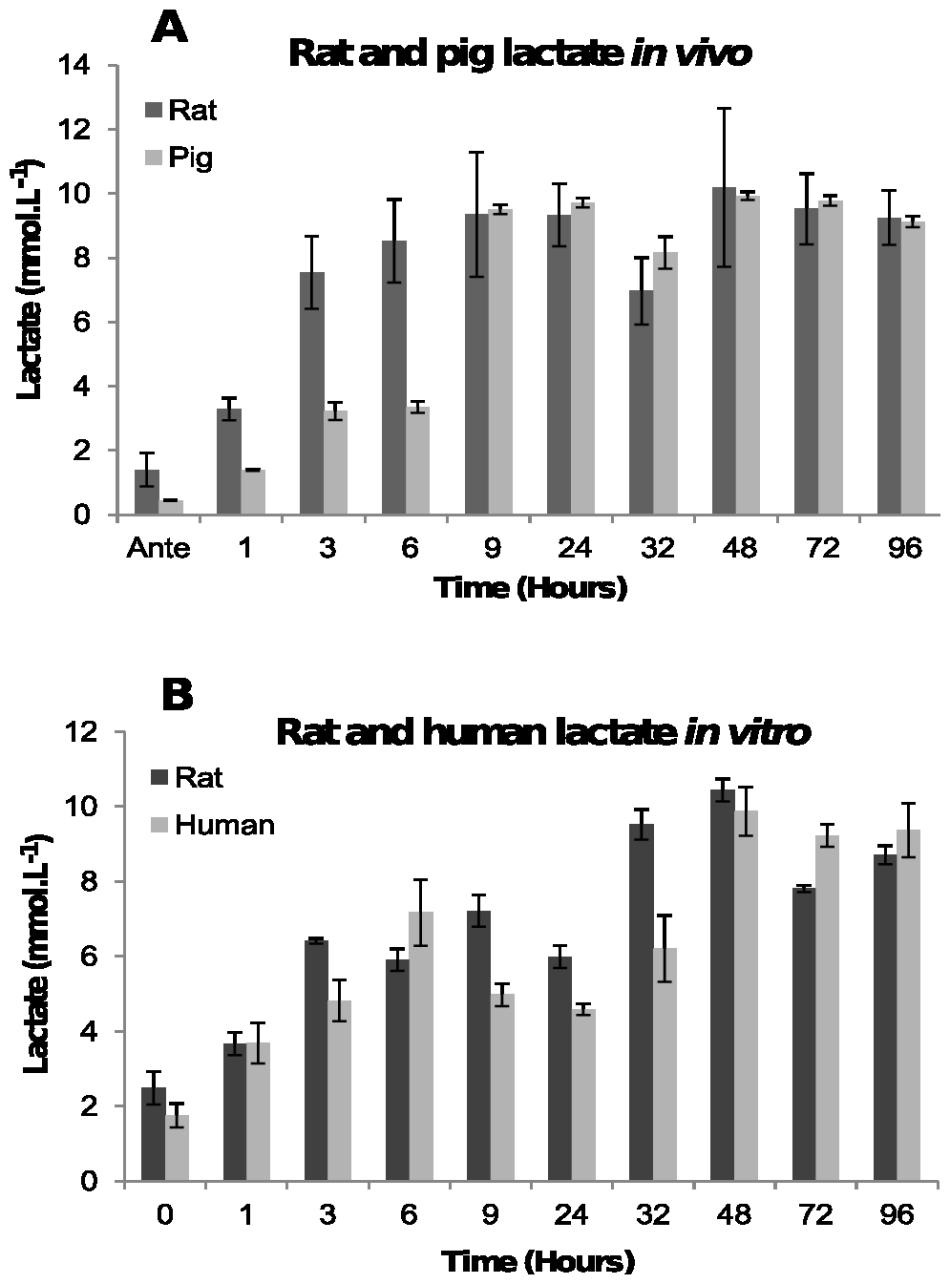
Concentration of lactate. [A] In post-mortem blood from rat and pig corpses over 96 hours. The average lactate concentration and the standard deviations in blood from seven rats and two pigs at each time point are shown. [B] In human blood and rat blood stored in a tube. The average concentration of lactate and the standard deviation at each time point from three human and two rat blood samples are shown.

In the *in vivo* experiments, (Figure 2 A) lactate concentration rose rapidly from between 0.5 and 2.5 mmol.L^-1^ immediately post-mortem to between 3 and 7.5 mmol.L^-1^ by three hours post-mortem. The lactate concentration reached a plateau by 9 hours, though a statistically significant decrease in rats at 32 hours was observed (*F* = 13.6, *p*-value < 0.000252) before increasing again. The lactate concentration in pigs also decreased at 32 hours, but it was not significant (*F* = 6.148, *p*-value < 0.0869).

Lactate concentrations also increased over time *in vitro* (Figure 2 B). The increase was not linear, with the lactate concentration showing a statistically significant decrease at 24 hours (F = 6.135, p-value < 0.035) before increasing again. 

The lactate concentrations after 96 hours are similar in both the *in vivo* and *in vitro* experiments, indicating that lactate concentrations are not the main driver of the pH differences seen in Figure 1. 

**Figure 3:**
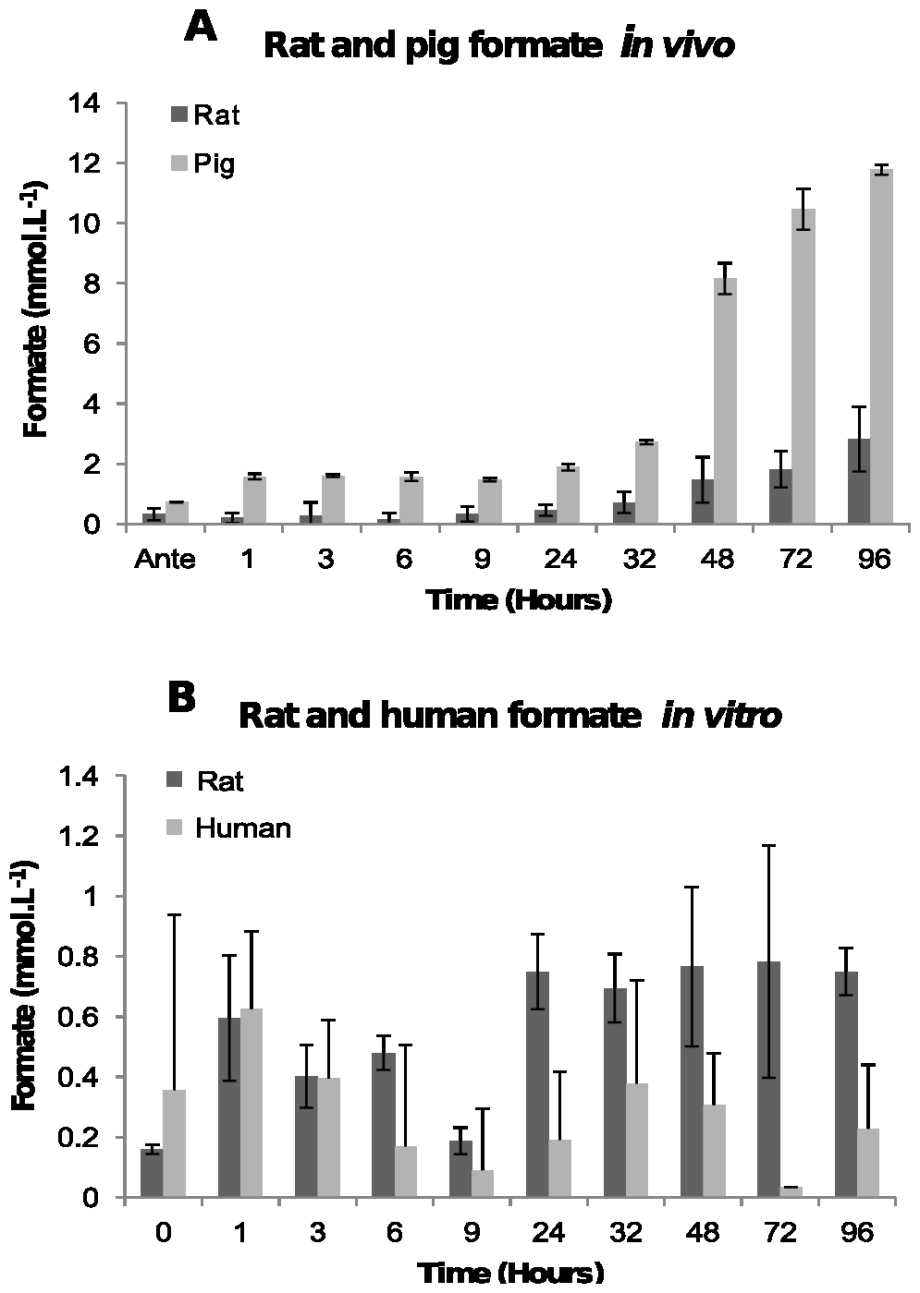
Concentration of formate. [A] In post-mortem blood from rat and pig corpses over 96 hours. The average formate concentration and the standard deviations in blood from four rats and two pigs at each time point are shown. [B] In human blood and rat blood stored in a tube. The average concentration of formate and the standard deviation at each time point from two human and two rat blood samples are shown.

The concentration of formate in blood *in vivo* remained fairly steady between 0.5 and 2 mmol.L^-1^ antemortem until 24 hours, after which it rose rapidly reaching a peak concentration of between 10.7 and 12.8 mmol.L^-1^ in pigs, and between 2 and 4 mmol.L^-1^ in rats by 96 hours post-mortem (Figure 3A). This is an increase of between 22- and 44-fold on the antemortem concentration.

Much lower concentration in *in vitro* samples (Figure 3B) was obtained. The concentration of formate was very variable but remained within antemortem range over the whole 96 hour time period. It is most likely that the variability in the samples is due to the limit of the assay, which is unable to accurately determine concentrations below about 1 mmol.L^-1^. 

Enzymes aldehyde dehydrogenase and formate dehydrogenase that produce formate only function at physiological pH. Aldehyde dehydrogenase is denatured outside the range of pH 6-10 [24] and formate dehydrogenase is denatured outside the range of 6-9 [25] and by 24 hours blood pH *in vivo* is less than 6 (Figure 1A) indicating that these enzymes will likely be inactive. Furthermore, NAD^+^ a cofactor required by these enzymes would not have been available. The increase in concentration of formate *in vivo* after 24 hours is most likely due to bacterial putrefaction.

The blood stored *in vitro* was sterile with no microbes present, consistent with the suggestion that microbial putrefaction cause the increases in formate concentration seen *in vivo*.

### Changes in NADH

NADH was examined to establish if large amounts are produced through metabolic pathways such as anaerobic glycolysis and peroxisomal catabolism of fatty acids. 

**Figure 4:**
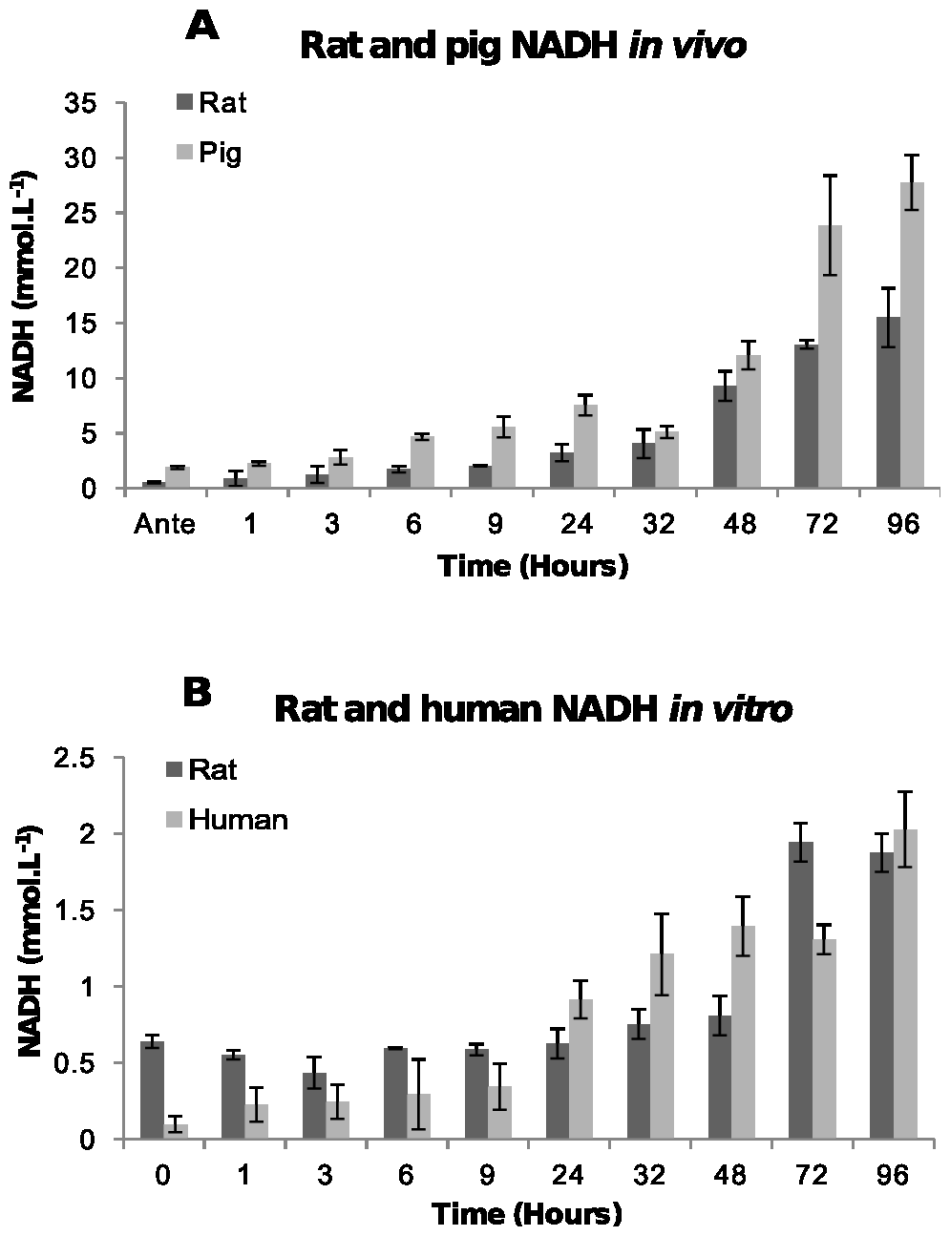
Concentration of NADH. [A] In post-mortem blood from rat and pig corpses over 96 hours. The average NADH concentration and the standard deviations in blood from six rats and two pigs at each time point are shown. [B] In human blood and rat blood stored in a tube. The average NADH concentration and the standard deviation at each time point from two human and two rat blood samples are shown.

In plasma NADH concentration increases rapidly and linearly after death between 1 and 2.5 mmol.L^-1^ at antemortem to between 15 and 28.5 mmol.L^-1^ by 96 hours post-mortem (Figure 4A). A statistically significant decrease (*F* = 47.75, *p*-value < 0.00532) at 32 hours was observed in both rats and pigs before increasing again. The increase in NADH concentration in pig blood is slightly higher than the NADH concentration in rat blood (Figure 4A). The reason for this has not been determined. The concentration of NADH in human blood and post-mortem rat blood *in vitro* increase slightly from 0.1 mmol.L^-1^ and 0.6 mmol.L^-1^ respectively at time zero to 2.3 mmol.L^-1^ and 2.0 mmol.L^-1^ by 96 hours, respectively (Figure 4B). The changes in NADH concentrations *in vivo* are significantly higher than the NADH concentrations *in vitro*. This is attributed to NADH being released from different tissues into the bloodstream as the tissues decompose, where blood in a tube has no other tissues which can release components into the blood. 

### Nitrogen-containing metabolites

The nitrogen-containing metabolites, ammonia, hypoxanthine and uric acid were examined to establish if the concentrations of these metabolites changed post-mortem, since these metabolites are produced in catabolic reactions involving limited or no oxygen.

**Figure 5:**
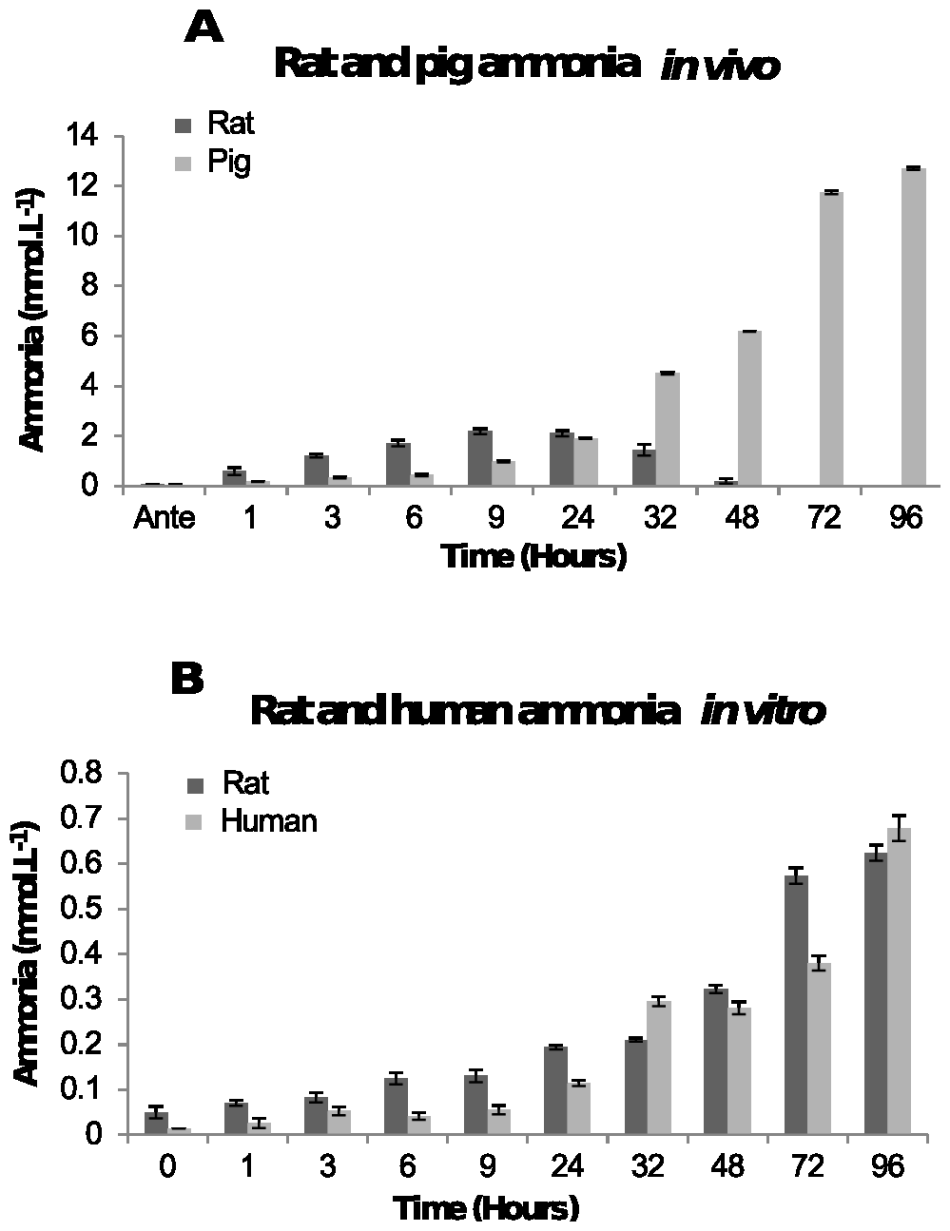
Concentration of ammonia. [A] In post-mortem blood from rat and pig corpses over 96 hours. The average ammonia concentration and the standard deviations in plasma from six rats and two pigs at each time point are shown. [B] In human blood and rat blood stored in a tube. The average ammonia concentration and the standard deviation at each time point from four human and two rat plasma samples are shown.

The concentration of ammonia in rat plasma post-mortem increased rapidly over the first 9 hours reaching the peak concentration of 2 mmol.L^-1^ before beginning to decrease until the concentration of ammonia was less than 11.7 μmol.L ^-1^, the sensitivity limit of the assay (Figure 5A). Figure 5A also shows that the pig plasma ammonia concentration increases after death from 0.26-0.48 mmol.L^-1^ at antemortem to 12-12.5 mmol.L^-1^ by 96 hours post-mortem. This is a 250-fold increase in the concentration of ammonia in the plasma. 

 The increase in ammonia concentration from human and rat blood stored in a tube (Figure 5B) was smaller over time from 0.013 mmol.L^-1^ (human) and 0.04 mmol.L^-1^ (rat) at time zero to 0.7 mmol.L^-1^ and 0.6 mmol.L^-1^ by 96 hours, respectively. 

The higher ammonia concentrations *in vivo* may be because proteins from different tissues may be released into the blood allowing more ammonia to be produced as those proteins are catabolised. In a blood tube there are no other tissues to release proteins into the blood as they degrade, so the concentration of ammonia is lower. 

**Figure 6:**
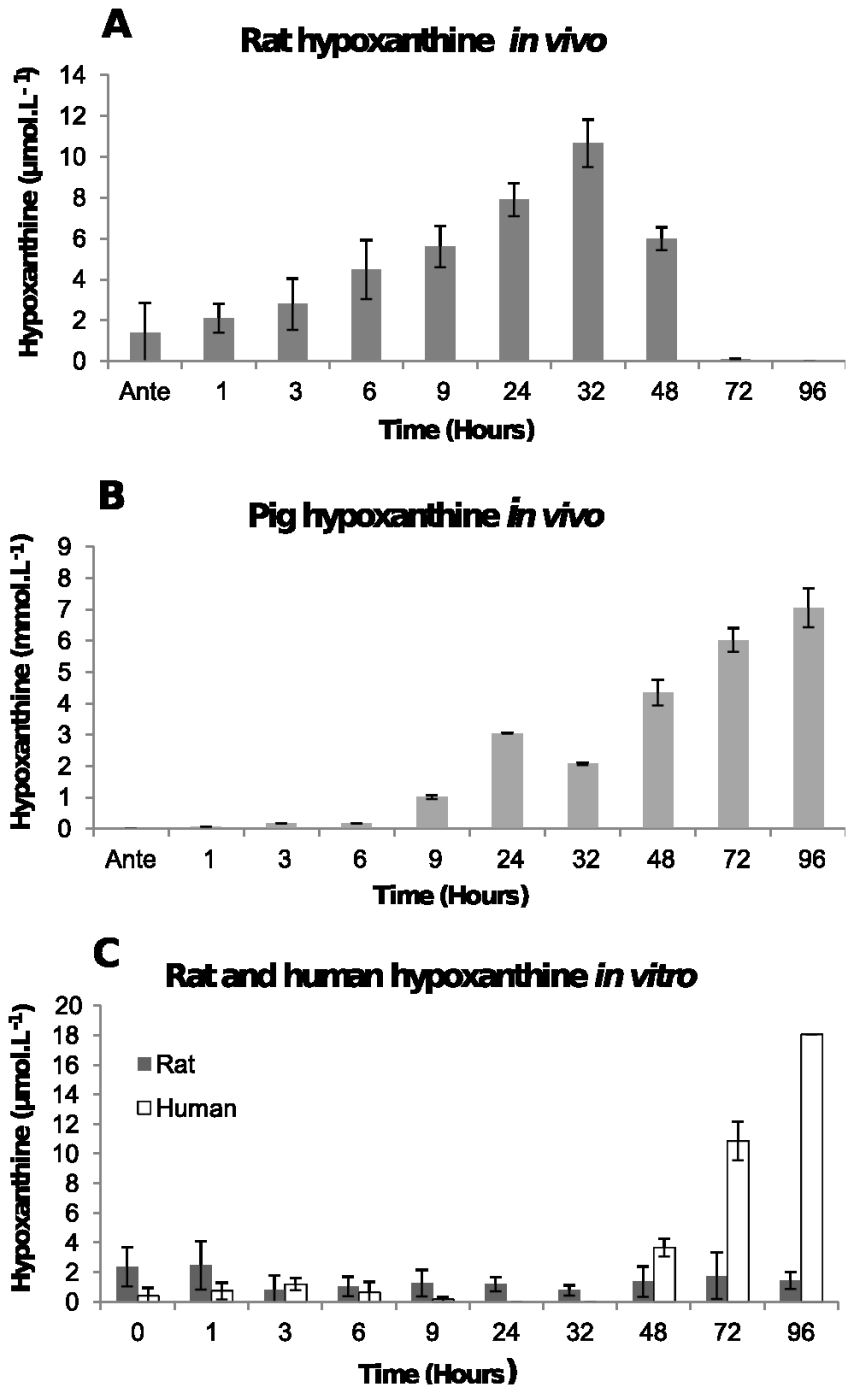
Concentration of hypoxanthine. [A] In post-mortem blood from rat corpses over 96 hours. The average ammonia concentration in plasma and the standard deviations from five rats at each time point are shown. [B] In post-mortem blood from pig corpses over 96 hours. The average hypoxanthine concentration in plasma and the standard deviations from two pigs at each time point are shown. [C] In human blood and rat blood stored in a tube. The average hypoxanthine concentration and the standard deviation at each time point from two human and two rat plasma samples are shown.

In the post-mortem rat plasma samples (Figure 6 A), the concentration of hypoxanthine increased steadily from less than 2 μmol.L^-1^ at antemortem to a maximum concentration of 11 μmol.L^-1^ at 32 hours, which is a 50-fold average increase. The hypoxanthine concentration then decreased and became undetectable by 96 hours. In contrast, pig plasma hypoxanthine concentration increased from 1.7 µmol.L^-1^ at antemortem to 6.5-7.5 mmol.L^-1^ by 96 hours post-mortem (Figure 6 B). This is a 1000-fold increase. 

In the human blood samples *in vitro* the concentration of hypoxanthine was less than 1 µmol.L^-1^ until 48 hours after which it increased to 18 μmol.L^-1^ by 96 hours (Figure 6 C) which is a 30-fold increase from antemortem concentrations. In contrast, the rat blood samples *in vitro* had concentrations that remained fairly steady (0.8-2 μmol.L^-1^) over 96 hours (Figure 6 C).

**Figure 7:**
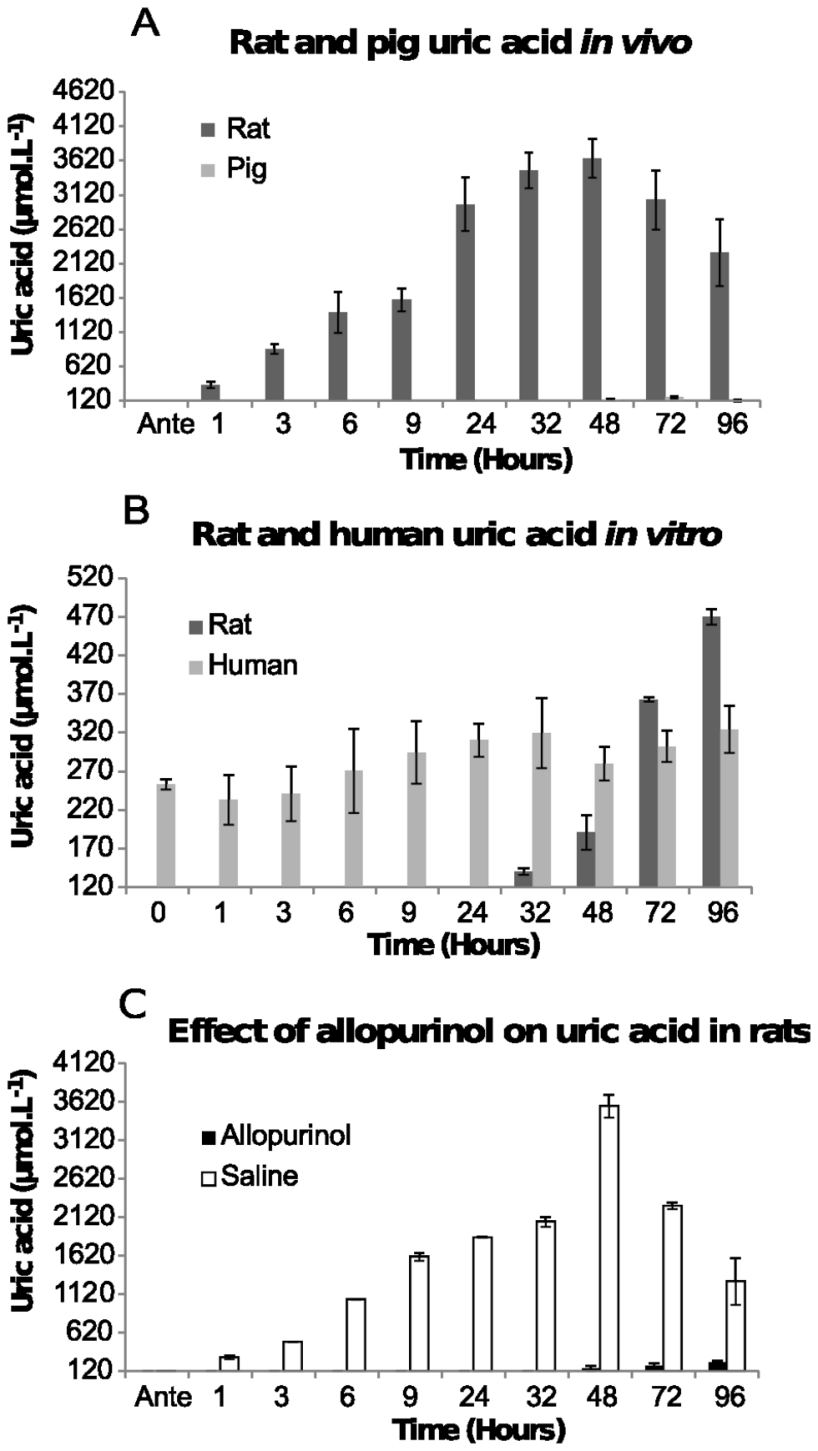
Concentration of uric acid. [A] In post-mortem blood from rat and pig corpses over 96 hours. The average uric acid concentration in plasma and the standard deviations from five rats and two pigs at each time point are shown. [B] In human blood and rat blood stored in a tube. The average uric acid concentration and the standard deviation at each time point from four human and two rat plasma samples are shown. [C] Affect of allopurinol on post-mortem rat uric acid concentrations. Three rats were administered allopurinol and one rat was given saline prior to euthanasia. The average concentration of uric acid is shown along with the standard deviations at each time point.

Uric acid concentration in a rat corpse increased significantly post-mortem from below the sensitivity limit of the assay (119 μmol.L^-1^) at antemortem to a peak of 4000 μmol.L^-1^ at 48 hours before decreasing (Figure 7A). This is in contrast to the results from the pig blood in which uric acid concentration was below the limit of detection (119 µmol.L^-1^) until 48 hours post-mortem when the uric acid concentration increased, but only to a maximum concentration of 170-180 µmol.L^-1^ which is just above the normal range (0-119 µmol.L^-1^) of uric acid in pig plasma [26]. 

 The uric acid concentration from rat blood *in vitro* remained below the limit of the assay until 48 hours when it increased reaching a maximum concentration of 450-500 μmol.L^-1^ by 96 hours (Figure 7B). The increase by 96 hours is a 5-fold increase from the normal range of uric acid in rat blood (29.75-119 μmol.L^-1^) [27]. Figure 7B also shows that uric acid in human blood *in vitro* remained relatively steady but low (between 200-500 μmol.L^-1^) for the whole 96 hours. The concentration did increase but the change remained within the normal range of uric acid in human plasma (137 µmol.L^-1^- 494 µmol.L^-1^).

Uric acid is synthesised by xanthine oxidase which requires oxygen, so was not expected to be active post-mortem. The striking increase in concentration of uric acid in rat blood was therefore unexpected. To confirm that xanthine oxidase was responsible for the increased concentration of uric acid, experiments were conducted on rats that were administered allopurinol (xanthine oxidase inhibitor) prior to euthanasia. The results clearly showed that uric acid synthesis was inhibited in the three rats that were administered allopurinol (Figure 7C). Saline was administered to one rat in place of allopurinol and uric acid concentrations were similar to those observed in Figure 7A. These data from rat plasma indicate that xanthine oxidase gives rise to the uric acid.

## Discussion

The aim of this study was to investigate metabolic changes that occur in blood post-mortem, with a view towards identifying biochemical markers that have potential for use in determining post-mortem interval. The results showed that blood pH and the concentrations of lactic acid, formate, NADH, ammonia, hypoxanthine and uric acid change post-mortem in both rat and pig corpses and in rat and human blood stored in blood tubes.

The most immediate biochemical change that occurred post-mortem is a fall in the concentration of oxygen due to absence of circulation, resulting in a switch to anaerobic metabolism with the absence of the citric acid cycle. Anaerobic glycolysis resulted in the accumulation of lactic acid and an increase in the concentration of NADH. Formic acid also increased post-mortem but because the production of this metabolite requires enzymes that function at physiological pH as well as NAD^+^ as a co-factor, the increase in formic acid is most probably attributed to bacterial putrefaction rather than anaerobic metabolism. It is unclear why the pigs and the rats had such large differences in the concentration of formic acid post-mortem. It may be because they possibly have different bacterial microflora. The accumulation of lactic acid and formic acid initiates the fall in pH post-mortem. Furthermore, because the circulatory system is stasis, the blood buffer systems fail resulting in a rapid pH decline as more acidic metabolites are produced. The speed of the plasma pH decrease was illustrated in Figure 1A where post-mortem blood pH fell from 7.45 to 6.0 within the first 24 hours after death. The plasma pH decrease is significant because it is thought to activate fibrinolysin enzymes which prevent blood clotting resulting in an increase in fluidity (non-coagulation) of the blood [28-31]. Although fibrinolysis was not examined in this study, the post-mortem blood remained fluid inside the rat and pig corpses over the 96 hour examination period, consistent with fibrinolysin enzymes being active. 

The difference seen in the blood pH *in vitro* compared to *in vivo* is due to two reasons. First, blood in a tube has no glucose store to supply fuel for anaerobic metabolism, consequently no significant lactate accumulation occurs to help lower blood pH. Secondly, erythrocytes do not contain lysosomes [32] so autolysis does not occur, meaning acidic cellular metabolites such as carbon dioxide, hydrogen ions, formic acid and lactic acid generated inside cells are not released rapidly from lysing cells to significantly lower pH as would occur in a corpse. Instead, the blood cells stored in a tube, slowly degrade over time due to cell membrane proteins not being maintained or replaced leading to slow cell lysis, rather than autolysis and therefore slow pH decreases compared to blood from a corpse.

The cause of the concentration decrease seen at 32 hours in the metabolite concentrations of lactate, NADH and hypoxanthine in post-mortem rat and pig blood, and the cause of the decrease seen at 24 hours in lactate *in vitro* is unknown. However, this decrease was very reproducible between all species tested.

The concentration of ammonia also increased significantly post-mortem, most strikingly by 250-fold in pig plasma. This is larger than the increase seen in post-mortem rat blood and in the *in vitro* experiments. The concentration of ammonia from amino acid and nucleotide catabolism in rats rose then declined possibly due to loss of ammonia by evaporation. It is possible that the reason for the difference in peak concentration reached between pig and rats is due to the putrefaction rate, since rats have complex gut microbiology [33] meaning putrefaction may occur faster, whereas the pigs were only 3-4 days old so would have less complex gut microbiology [34] having a slower rate of putrefaction due to less bacterial diversity. The *in vitro* experiments show a similar profile to the pig except that the concentration of ammonia was significantly less, suggesting that either the ammonia is lost from the blood tube or some ammonia in the corpse is made from non-blood tissue. Further studies are needed to determine if the rat versus pig difference is due to differences in the rate of ammonia loss or metabolic differences between the species.

Hypoxanthine is oxidised by xanthine oxidase to uric acid which is the end product of purine metabolism in humans [17]. The xanthine oxidase enzyme in mammals is present in the liver and the small intestinal mucosa. Since there is no xanthine oxidase in the blood, the end product of nucleotide catabolism in the blood is therefore hypoxanthine rather than uric acid. This is what was seen in the human *in vitro* experiments and the pig *in vivo* experiments, which showed that the concentration of hypoxanthine increased, whereas the uric acid concentration remained steady. The concentration of hypoxanthine and uric acid in the rat *in vivo* experiments was in contrast to the human and the pig concentrations. The rat hypoxanthine and uric acid concentrations both increased and then slowly decreased. A reason for this difference may be because rats have a high concentration of lung xanthine oxidase [35,36]. Rat lung xanthine oxidase is able to diffuse into the bloodstream during hypoxia where it clears hypoxanthine rapidly from the plasma [35,36] by converting it into uric acid, which is what the hypoxanthine and uric acid experiments showed. Further support showing that the xanthine oxidase was indeed giving rise to the uric acid in the rats was seen in the allopurinol experiment. This was an unexpected finding as xanthine oxidase requires oxygen as a cofactor to convert hypoxanthine and xanthine to uric acid [16]. Exactly how this reaction is able to continue post-mortem is not known and requires further study.

Differences were also seen in the rat between the *in vitro* and *in vivo* experiments. As decomposition progresses in a corpse, purines from blood cells as well as different tissues are being released into the extracellular fluid, and are then converted into uric acid in the plasma over the 96 hour period. In rat blood stored in a tube, the uric acid does not increase until 48 hours. The reason for this delay is most likely because of decomposition. For example, previous research has shown that it takes 48 hours for white cells to start to decompose and release their contents after death [37]. Therefore, because it takes 48 hours for the white cells to lyse and this is the main purine source available to xanthine oxidase in a tube, a constant uric acid concentration is maintained until the white cells start to lyse releasing their purines.

This study used post-mortem blood from rats and pigs due to the practical difficulties of obtaining post-mortem human blood samples and it is not known how exactly such data could be translatable to human cadavers. Furthermore, post-mortem research using both pigs and humans found that model organisms are preferential to human cadavers for replicated studies because of the vast differences between individual human cadavers, and the ability to be able to control many of the variables influencing decomposition rates with animals [38]. No post-mortem human blood samples were used; instead, we used antemortem human blood samples. The metabolic changes that occur in these samples had similarities, though also some important differences, to changes that occurred in the pig and the rat. This indicates that findings of this research may be translatable to human cadavers. However, further work will need to be done using post-mortem human blood to directly analyse metabolic changes that occur in humans post-mortem and whether the biomarkers identified in this study could be used in humans to estimate PMI. Estimating PMI is the most sought after piece of information associated with a death investigation [39] and biochemical methods are currently being explored as an approach for PMI estimation. Of the markers investigated here, hypoxanthine, ammonia and NADH currently hold the greatest promise as PMI indictors as this study showed that their concentrations are increased linearly post-mortem, these metabolite changes are not produced by anaerobic bacteria during putrefaction, so putrefaction does not significantly effect the production of these metabolites and they can be very quickly measured and analysed via enzymatic assays. Therefore, this study has shown that biochemical markers, namely metabolites extracted from post-mortem plasma have the possibility of providing a reliable estimate regarding time since death. However, despite the metabolites examined here having potential as possible PMI markers, more research is needed to understand the affect that different temperatures and humidity have on these post-mortem metabolite concentrations, what concentrations these metabolites reach in the bloodstream after more than 96 hours and if these metabolite concentrations are comparable to post-mortem human blood samples before they will be able to be used to estimate PMI.

## Materials and Methods

### Ethics permissions

This study was carried out in strict accordance with the NZ Animal Welfare Act 1999 in the University of Otago Code of Ethical Conduct for the Use of Animals. The protocols were approved by the Animal Ethics Committee of the University of Otago (Permit Numbers: ET-25/10 and ET 21/12) for the euthanasia of the animals for the purpose of this study. Human blood samples were collected from healthy participants after informed written consent was obtained. Ethical permission approving the study was granted from the University of Otago Human Ethics Committee (Permit Number: 10/168) along with the participant information sheet and consent forms.

### Sampling procedures

Twenty adult Sprague-Dawley rats weighing between 400 and 500g and two white domestic 3-4 day old pigs weighing 1.1 and 1.7 kg respectively, were euthanised by cervical dislocation and kept in an incubator at constant conditions (22°C, 32-46% relative humidity). Before euthanasia the animals were sedated with subcutaneous injections of Ketamine and Domitor (75 mg/kg of Ketamine and 0.5 mg/kg of Domitor for rats and 20 mg/kg of Ketamine and 0.2 mg/kg of Domitor for pigs) to enable an antemortem blood sample to be obtained via cardiac puncture. Within 10 minutes following death, the animals were dissected open and a blood cannula inserted into the abdominal aorta, for repeated blood samplings over a 96 hour post-mortem time period.

For post-mortem blood stored in a tube, once rats were euthanised, 5 mL of blood was removed via cardiac puncture using a needle and syringe and placed into a 5 mL Ethylenediaminetetraacetic acid (EDTA) venapuncture collection tube. The venapuncture tube was then placed into the incubator alongside the animal corpses and sampling carried out over the 96 hour post-mortem time period.

Human blood was collected by a trained phlebotomist into 5 mL EDTA venapuncture collection tubes. The 5 mL EDTA blood tube was placed in the incubator with the animal corpses and blood sampled over the 96 hour time period.

### Blood and plasma preparations

 At each sampling time post-mortem, 500 μL of human blood or rat blood was collected from a tube, and 500 μl of rat blood and 2 mL of pig blood were collected from the animal corpses and the samples placed into clean microcentrifuge tubes. Samples were centrifuged at 13000 rpm for 10 minutes to obtain plasma for metabolite analysis, except for lactate assay’s in which whole blood was used. The lactate and formate assays required deproteination of the plasma and whole blood samples prior to analysis. This was carried out using the method described in [40] which also oxidises any endogenous NADH to NAD^+^.

### Metabolite assays

Blood pH was measured using a micro-electronic pH meter (ISFETCOM) Model S2K712 as only 20 µl was used. Certified calibration buffer standards (ISFETCOM) were used before each pH analysis.

The concentration of lactate was measured using a spectrophotometric method [41] with slight modifications, samples being incubated for 40 minutes instead of 30 minutes and concentration calculated using a standard curve rather than the Beer-Lambert law calculation. The concentration of NADH in blood samples (100 μl) was measured using a spectrophotometric method based on the absorption of NADH at 340 nm [42], in conjunction with a standard curve. 

The concentration of formate in the blood was measured using a spectrophotometric method based on determination of NADH using formate dehydrogenase to catalyse the oxidation of formate to carbon dioxide, coupled to the reduction of NAD^+^ to NADH [43]. Slight modifications were made to the method [43], in which samples were measured every 30 seconds for 5 minutes, instead of after 2 minutes and concentration calculated using a standard curve rather than the Beer-Lambert law calculation. 

The concentration of hypoxanthine in samples was measured using an enzymatic Fluorescence Assay Kit, Amplex Red® xanthine oxidase assay Kit (Invitrogen, Cat no. A22182) following the manufacturer’s instructions with slight modifications, samples being measured at 60 minutes instead of 30 minutes. 

Ammonia concentration was measured using an enzymatic ammonia assay kit, (Sigma-Aldrich, Cat no. AA0100-1KT) following the manufacturer’s instructions. 

Uric acid concentration was measured using a Reflotron meter and uric acid strips (Roche Diagnostics, Cat no. 10745103202) according to the manufacturer’s instructions.

All analytical methods, which are based on established biochemical protocols, were validated by using blood or plasma to which varying amounts of target metabolites had been added.

### Statistical analysis

Average concentrations and standard deviations were calculated using Microsoft Excel 2009 and Analysis of Varience (ANOVA) followed by a post-hoc Tukey analysis performed using R studio (www.r-project.gov) with a p-value smaller than 0.05 being significant.
